# Investigating the residual effect of silver nanoparticles gel as an intra-canal medicament on dental pulp stromal cells

**DOI:** 10.1186/s12903-022-02542-2

**Published:** 2022-11-30

**Authors:** Ahmed Mahmoud, Sybel Moussa, Rania El Backly, Reem El-Gendy

**Affiliations:** 1 Endodontics, Faculty of Dentistry, Kafr El-Sheikh University, Kafr El-Sheikh, Egypt; 2 Division of Oral Biology, University of Leeds, School of Dentistry, Leeds, UK; 3 Endodontics, Conservative Dentistry Department, Faculty of Dentistry, Alexandria University, Alexandria, Egypt; 4 Endodontics, Conservative Dentistry Department and tissue engineering laboratories, Faculty of Dentistry, Alexandria University, Alexandria, Egypt; 5 Faculty of Dentistry, Suez Canal University, Ismailia, Egypt

**Keywords:** Regenerative endodontic procedures, Dental pulp stromal cells, Intra-canal medicaments, Silver nanoparticles, Calcium hydroxide

## Abstract

**Background:**

The aim of this study was to evaluate the indirect effects of residual silver nanoparticles (AgNPs) gel on human dental pulp stromal cells (DPSCs).

**Methods:**

Ninety-five dentin discs (4x4x1 mm) were prepared from freshly extracted human single-rooted teeth following institutional ethical approval and informed consent. Samples were cleaned, autoclaved, and treated with: 1.5%NaOCl, Saline and 17% EDTA then randomly assigned to 5 groups that received 50 μl of one of the following treatments: 0.01%AgNPs, 0.015%AgNPs, 0.02%AgNPs, Calcium hydroxide (Ca (OH)_2_) or no treatment for 1 week. Discs were washed with Saline and 17%EDTA then seeded with DPSCs and incubated for 3 and 7 days. At 24 hours unattached cells were collected and counted. At each time point cytotoxicity (LDH assay), cell viability (live/dead staining and confocal microscopy) and cell proliferation (WST1 assay) were assessed. All experiments were repeated a minimum of 3 times using DPSCs isolated from 3 different donors for each time point assessed (*n* = 9/group). Statistical analysis was done using One-Way ANOVA followed by Tukey’s test and Kruskal Wallis followed by post-hoc comparisons with significance set at *p* ≤ 0.05.

**Results:**

After 24 hours, the percentage of DPSCs attachment ranged between 92.66% ±4.54 and 95.08% ±1.44 with no significant difference between groups (*P* = 0.126). Cell viability was ≥92% at 24 hours for all groups. However this percentage dropped to less than 60% at 3 days then started to rise again at 7 days. There was no significant difference in cytotoxicity between different groups at all time points except for 0.01%AgNPs group which had the highest cytotoxicity. DPSCs proliferation increased significantly from 3 to 7 days in all groups except for Ca (OH)_2_ which showed lower proliferation rates at both 3 (45.89%) and 7 days (79.25%).

**Conclusion:**

Dentin discs treated for 7 days with concentrations of AgNPs gel (0.01–0.02%) allowed more than 90% DPSCs cell attachment after 24 hours. The cytotoxicity and proliferation of DPSCs in response to AgNPs gel were comparable to those with calcium hydroxide. This suggests that AgNPs gel may represent a promising future candidate for clinical use in regenerative endodontics. However, its effects may be concentration-dependent warranting further investigation.

**Supplementary Information:**

The online version contains supplementary material available at 10.1186/s12903-022-02542-2.

## Background

The protocol for regenerative endodontic procedures (REPs) has been continuously changing since coining of the term in 2007. Since then, both the European Society of Endodontology and the American Association of Endodontists have been updating the current clinical protocol of treatment due to continuous advancements in the field [1, 2]. Indeed, two of the most challenging issues facing REPs are the adequacy of disinfection and biocompatibility of intra-canal medicaments and their residues with viable recruited stem cells. This is particularly important as the field crosses over to the treatment of the mature necrotic permanent tooth as well [3, 4].

Recent studies have shown that, persistent infection could negatively impact the regeneration process [5] and that most failed cases following REPs can be attributed to the presence of residual infection [6]. Additionally, persistent infection can also negatively affect root development [7]. Therefore, it is of prime importance to eradicate intra-radicular infection using intra-canal medicaments before the occurrence of any regenerative process [8]. These medicaments should possess adequate antibacterial properties and provide a favorable environment for stem cells attachment, proliferation, and differentiation. Most studies published, so far, in regenerative endodontics have used either calcium hydroxide or triple antibiotic paste [9].

Hoshino et al., developed the triple antibiotic paste (TAP) consisting of metronidazole, ciprofloxacin, and minocycline, which has been widely used in cases of REPs because of its effectiveness in disinfecting the root canal space once occupied by infected necrotic pulp [10]. Double antibiotic paste (DAP) which consists of metronidazole and ciprofloxacin without minocycline has also been proposed for REPs. When there is sensitivity to one of these antibiotics, calcium hydroxide (Ca (OH)_2_) was the next logical choice [11] due to its highly alkaline pH (approximately 12) which can disinfect the root canal space and stimulate hard tissue repair [12].

The size of an adult pulp is less than 100 μm [13], which may falsely indicate the ease of its regeneration. However, REPs are highly subjected to failure due to persistent infections when using lower TAP and DAP concentrations [14]. Hence, higher concentrations of TAP, DAP (1–5 mg/ml) and Ca (OH)_2_ are recommended to eradicate all the intra-canal infection, but with the risk of adverse effects on the physical [15], mechanical [16] and chemical properties [17] of radicular dentin. In addition, higher concentrations of TAP are detrimental to the survival of apical papilla stem cells [18, 19]. The possibility of staining the tooth structure [20] and more importantly the development of antibiotic resistance [21] are also considered limitations for the use of TAP and DAP.

Therefore, the search for the ideal intra-canal medicament continues in an attempt to realize the balance between providing adequate and residual antibacterial effect with maintaining a hospitable niche for stem cells to regenerate the lost or damaged tooth structures [22]. Recently, alternative antimicrobials such as Silver Nanoparticles, calcium hypochlorite, chitosan-based medicaments, bioactive glass and others [22, 23] have been suggested.

Silver nanoparticles (AgNPs) have been proposed as antibacterial agents for intra-canal disinfection due to their broad-spectrum and highly efficient antimicrobial activities [24, 25]. The antimicrobial effect of AgNPs as an irrigant and intra-canal medication gel against *E. faecalis* biofilms was investigated and it was found that 0.02% AgNPs gel significantly disrupted the biofilm compared to 0.01% AgNPs gel [26]. Sadek et al. also investigated the antimicrobial effect of 0.02% AgNPs gel as an intra-canal medicament and found that it can effectively kill 99.4% after 24 hours and 99.9% after 7 days of 3 weeks old E.faecalis biofilm [27]. Ag + released from AgNPs usually generates reactive oxygen species (ROS) causing oxidative stress after entering into the bacterial cells [28]. As ROS levels increase, the glutathione (GSH) level decreases and at the same time lactate dehydrogenase (LDH) increases in the medium, then induces apoptosis [29]. While these results are promising, the use of silver nanoparticles gel for regenerative endodontic applications has still not been validated.

Since available information is still limited about the cytocompatibility of the AgNPs gel as an intra-canal medicament, the aim of this study was to investigate the effect of different AgNPs gel concentrations on viability, attachment and proliferation of human dental pulp stem cells (DPSCs) for potential use as an intra-canal medicament for REPs. The null hypothesis was that, different concentrations of AgNPs would have comparable results to Ca (OH)_2_ on viability, attachment and proliferation of DPSCs.

## Methods

### AgNPs preparation

AgNPs in methyl-cellulose (Nanotech, Cairo, Egypt) (Supplementary Fig. 1) with different concentrations 0.01, 0.015 and 0.02% were prepared by chemical reduction method using sodium borohydrite as reducing agent of silver nitrate (AgNO3) solution in water medium with polyvinylpyrrolidone (PVP) as stabilizing agent [30]. For the synthesis of silver nanoparticles, 10 ml of 1% ethanolic solution of PVP and 0.2 ml of 0.1 M silver nitrate powder were taken in a closed test tube containing 25 ml of sterile water and placed in microwave oven, the procedure was operated at 100% power of 1000 W and frequency 2450 MHz for 5 seconds. The colorless solution turned to the characteristic grayish yellow color, indicating the formation of silver nanoparticles [31].

### Development of the in-vitro model using DPSCs and treated dentin discs

#### Preparation of dentin discs

Freshly extracted human single-rooted teeth due to periodontal reasons without caries, resorption, or fracture were provided by the Oral Surgery Department, Faculty of Dentistry, Alexandria University following institutional ethical approval (IRB NO: 00010556 – IORG 0008839) (3/2020) and informed patient consent. Teeth were collected and stored in physiological saline at 4 °C. Ninety-five radicular dentin discs (4x4x1 mm) were obtained from these teeth by sectioning off the crown and the apical 3 mm from the root using a low-speed diamond disc (MICRODONT, Lda, Brazil) under water coolant. Soft tissues and cementum were removed from the outer tooth surface then the roots were vertically sectioned along the mid-sagittal plane into two halves using a low-speed diamond saw (Isomet, Buehler, Lake Bluff, IL, USA) under constant irrigation obtaining 1 dentin disc from each half [32]. The discs were then sonicated in deionized water for 5 min and maintained in 100% humidity at 4 °C. Before use, dentin discs were sterilized for 20 minutes at 121 °C using a high-pressure steam autoclave.

#### Dentin surface pre-treatment

The pulpal surface of each dentin disc was irrigated using the following protocol: 1 mL of 1.5% NaOCL (Milton solution, Milton, UK) for 5 mins, 3 ml saline (Baxter Health Care, Thetford, UK) for 3 mins (1 ml / 1 min), 1 mL of 17% EDTA (Bio-world, Dublin, OH, USA) for 5 mins then 3 ml saline for 3 mins (1 ml/ 1 min) [33]. Discs were individually placed into sterile 48-well plates (Costar, Corning, NY, USA) with the pulpal surface facing upwards and randomly allocated to either one of the 5 groups; 3 treatment and 2 control groups (*n* = 19 per group). Discs in the three treatment groups were treated with either 50 μL of 0.01% AgNPs gel, 0.015% AgNPs gel or 0.02% AgNPs gel. Dentin discs of the positive control group were treated with 50 μL of Ca (OH)_2_ (UltraCal, Ultradent, South Jordan, UT, USA) and in the negative control group received no treatment. All dentin discs were then incubated for 7 days at 37 °C, 100% relative humidity in a 5% CO_2_ incubator (MCO-20AIC, Sanyo, Osaka, Japan). After the incubation period, samples from all groups were rinsed with 3 mL of saline to remove the medicaments. Then, the pulpal surface of each dentin disc in all groups was irrigated with 1 mL of EDTA 17% for 5 mins followed by 3 ml saline for 3 mins (1 ml / 1 min). To ensure complete removal of medicaments from the dentin surface, the medicament layer was gently peeled off from the surface of each dentin disc before EDTA irrigation. One sample from each group was imaged using scanning electron microscopy (SEM) (Science Systems Ltd., Tokyo, Japan) to ensure the complete removal of medicaments after the irrigation protocol (total number of samples = 5). The remaining ninety dentin discs received a rinse with 500 μL of phosphate-buffered saline (PBS) (BioWhittaker, Walkersville, MD, USA) and were transferred into individual wells of a sterile non-treated low-attachment 24-well plate (Costar, Corning, NY, USA) with the treated pulpal surface facing upwards.

#### DPSCs culture and seeding on treated dentin

DPSCs were provided by the division of Oral Biology, Leeds School of Dentistry. Cells were isolated from intact third molars extracted after patient’s informed consent and under the ethical approval of Leeds dental and skeletal tissue bank (DREC number 170619/NH/277). DPSCs from 3 different donors stored at − 80 °C were thawed and cultured in Alpha Modified Eagle’s Minimum essential medium (α-MEM) (BioWhittaker, Walkersville, MD, USA) supplemented with 20% Fetal Calf Serum, 1% L-glutamine and 1% penicillin/streptomycin (complete media) then grown to 80% confluency. Ten thousand sub-confluent DPSCs (passage 4–6) were seeded on the treated pulpal surface of each dentin disc. Seeded samples were then incubated for 3 days (*n* = 45) and 7 days (*n* = 45) at 37 °C and 5% CO_2_. All experiments were carried out between passages 4–6 and were repeated a minimum of 3 times using DPSCs isolated from 3 different donors for each time point assessed (*n* = 9/group).

### Assessment of DPSCs attachment to dentin disc surface

#### Calculating percentage of DPSCs attachment to dentin surface after 24 hours

After a 24-hour attachment period, the media containing the unattached cells was used to determine the number of attached DPSCs on the dentin discs (*n* = 18/group). The media from each individual well containing unattached cells was transferred into a sterile Eppendorf tube and centrifuged to form a cell pellet which was resuspended in 30 μL of media. The resuspended cells were stained with trypan blue and counted using a hemocytometer. The number of attached cells was calculated by subtracting the number of unattached cells from the total cell number seeded (10 × 10^3^ cells/well). Each dentin disc was then irrigated with 100 μL of media. Then 500 μL of complete media was added to each well and all samples were incubated to complete 3 and 7 days from the initial time of DPSCs culture.

#### Confirming DPSCs attachment to dentin surface using SEM imaging

After 3 and 7 days of culture, 3 dentin discs from each group were processed for scanning electron microscopy (SEM) to evaluate the morphology of DPSCs (*n* = 3/ group). Briefly, dentin discs were gently washed with PBS to remove unattached DPSCs, fixed with 10% neutral buffered formalin, dehydrated through freeze drying, sputter coated with gold then images were taken using a HITACHI S-3400 N scanning electron microscope (Science Systems Ltd., Tokyo, Japan) at a magnification of × 500 and × 2000.

### Assessment of cytotoxicity of AgNPs using lactate dehydrogenase (LDH) cytotoxicity assay

In the current study, the lactate dehydrogenase (LDH) cytotoxicity assay was used to evaluate the cytotoxicity of the different concentrations of AgNPs on DPSCs. The release of LDH from DPSCs was measured using LDH-Cytotoxicity Assay kit-II (BioVision, California, USA) in supernatant media collected after 24 hours (*n* = 18/group), 3 days (*n* = 9/group) and 7 days (*n* = 9/group) of culture. In summary, 25 μL of the collected media at the different time points were transferred into a sterile 96-well plate and mixed with 100 μL of LDH reaction solution prepared according to the manufacturer’s protocol and incubated for 30 min at 37 °C away from light. Maximum LDH release (high control) was obtained by adding 10 μL of the provided lysis solution (BioVision) to 10 × 10^3^ cells cultured in monolayer (*n* = 3) to provide total cell death giving the maximum release of LDH. The complete culture media was used as low control/negative control. A microplate reader (BioTek, Winooski, VT, USA) was used to quantitatively measure the colorimetric change at a wavelength of 450 nm in samples and controls. The percentage of cytotoxicity in the different treatment groups was calculated according to the following equation: cytotoxicity (%) = (experimental absorbance value - low control absorbance value) / (high control absorbance value - low control absorbance value) × 100.

### Assessment of DPSCs viability using the LIVE/DEAD staining assay

A LIVE/DEAD staining assay (Molecular Probes, Invitrogen, Eugene, OR, USA) was used to assess viability of DPSCs on dentin disc surfaces. The LIVE/DEAD stain comprised of calcein-AM for staining of live cells and ethidium homodimer (EthD-1) for dead cells. Excitation and emission values for calcein and EthD-1 are 494/517 nm and 528/617 nm, respectively. Briefly, 20 μL EthD-1 and 5 μL of calcein AM were mixed with 10 mL of sterile plain media without any additives. The LIVE/DEAD working solution was added to each DPSCs seeded dentin disc, followed by incubation at room temperature for 1 hour away from light. Immediately after staining, samples were imaged using a confocal scanning microscope (Leica DM6 M, Wetzlar, Germany) at 10X magnification using Leica Application Suit X (LAS X) software, v. 3.5.7.23225. At least 10 images per group were acquired.

### Assessment of DPSCs proliferation on dentin using WST-1 assay

After 3 and 7 days of culture, DPSCs proliferation on the treated dentin discs was evaluated using a cell proliferation reagent WST-1 (Roche Applied Science, Penzberg, Germany). Briefly, dentin discs at 3 days (*n* = 9/group) and at 7 days (*n* = 9/group) were washed with 200 μL of α-MEM, incubated in a mixture of 100 μL of plain α-MEM without any additives and 10 μL of WST-1 reagent for 4 hours at 37 °C and 5% CO_2_. Ten μL of WST-1 reagent was added to 10 × 10^3^ newly counted cells suspended in 100 μL of α-MEM (*n* = 3) to obtain maximum WST-1 signal (high control). Furthermore, WST-1 reagent was also added to wells containing 100 μL of α-MEM with no cells (low control). Following incubation, 100 μL of the reaction mixture was transferred from each well into another sterile 96-well plate and absorbance values were read at 450 nm. The percentage of DPSCs proliferation on treated dentin discs was calculated using the following equation: Proliferation of attached DPSCs (%) = (experimental absorbance value - low control absorbance value) / (high control absorbance value - low control absorbance value) × 100. The percentage of cell proliferation of untreated cells was considered 100%.

#### Statistical analysis

Descriptive statistics were performed. Normality was tested using Shapiro Wilk test. Attachment, proliferation and cytotoxicity after 3 and 7 days showed normal distribution while cytotoxicity after 24 hours was not normally distributed. Comparisons between groups were done using One Way ANOVA (for normally distributed results) followed by Tukey’s test with Bonferroni adjustment and Kruskal Wallis test (for non-normally distributed results) followed by post hoc comparisons with Bonferroni correction. Comparisons of cytotoxicity between the three-time points for each group were done using Kruskal Wallis test due to independency of specimens at each time point. Differences in proliferation percentage between two time points were compared using independent t test. The level of statistical significance was set at *p* ≤ 0.05. Data were analyzed with IBM SPSS statistical software V.25, (SPSS Inc).

## Results

### Characterization of AgNPs

**Fig. 1:**
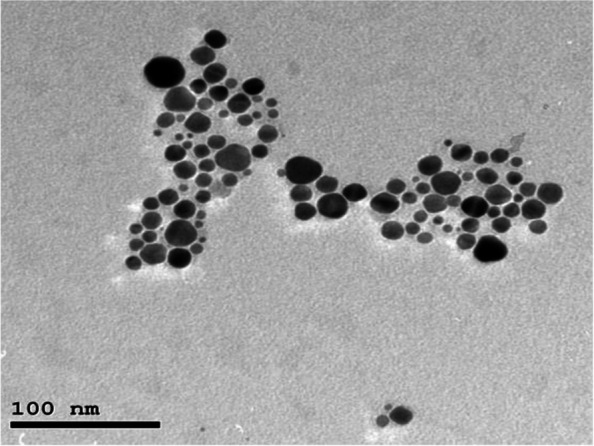
Transmission electron microscopy image of AgNPs showing their spherical shape and size range (16 ± 2 nm)

Synthesis of AgNPs in nanoscale was verified by ultraviolet visible spectroscopy (Agilent Technologies, Waldbronn, Germany) that monitored the absorption spectra of the formed AgNPs at a wave length that ranged between 300 and 700 nm with maximum wave length equal to 410 nm, which coincided with the optical properties of nanoparticles [34]. Also, the size and shape of AgNPs were identified through a high-resolution transmission electron microscope (JEOL JEM-2100, JEOL, Tokyo, Japan) at an accelerating voltage of 200 kV, which indicated the spherical shape of particles with average size 16 ± 2 nm (Fig. 1).

### Verifying the effectiveness of the irrigation protocol in removing of medicaments

**Fig. 2:**
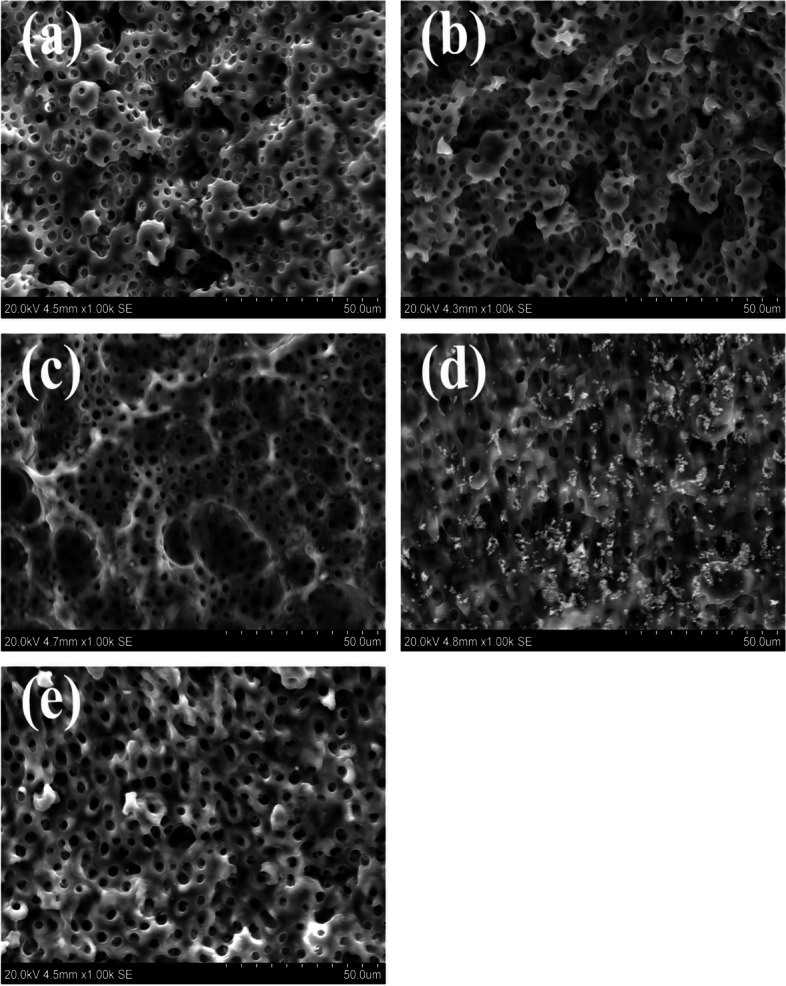
SEM images of the dentin surface showing one representative sample from each treatment group after finalizing the irrigation protocol to confirm complete removal of the medicaments: 0.01% AgNPs gel in (**a**); 0.015% AgNPs gel in (**b**); 0.02% AgNPs gel in (**c**); Calcium hydroxide group in (**d**) showing remnants of Ca (OH)_2_ particles still adherent to the surface and Non-treated dentin control group (**e**). (Magnification × 1000)

Representative SEM images demonstrated complete removal of all three concentrations of AgNPs gel from dentin surfaces while calcium hydroxide showed some remnants attached to the dentin surface (Fig. 2).

### DPSCs attachment and morphology on dentin surface

**Fig. 3:**
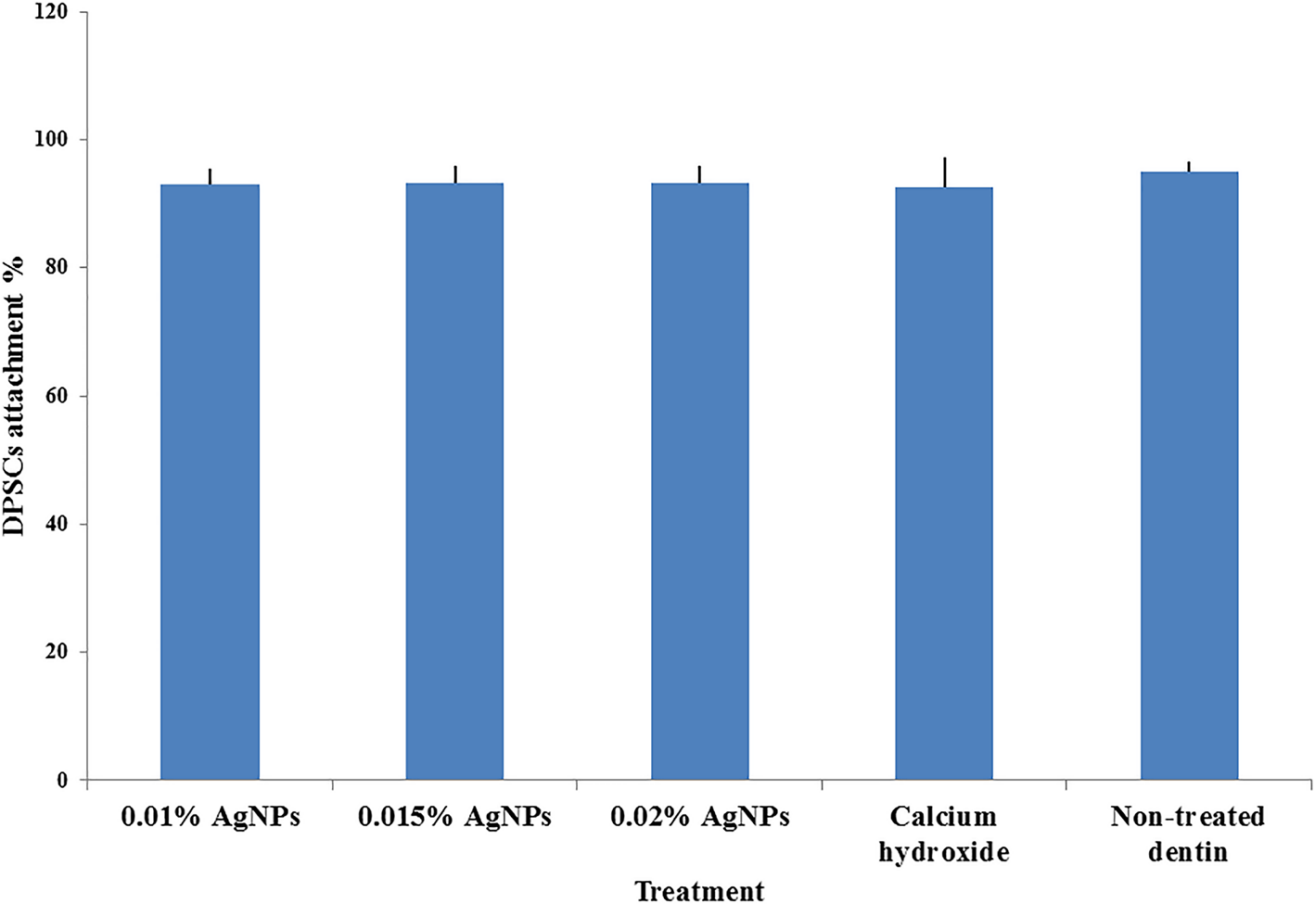
The percentage of DPSCs attachment to dentin surfaces in the different groups (mean ± SD): 0.01% AgNPs, 0.015% AgNPs, 0.02% AgNPs, Ca (OH)_2_ and non-treated dentin. There was no statistically significant difference in DPSCs attachment on dentin surfaces between any of the groups

The minimum percentage of cell attachment was 92.66% ±4.54 in the Ca (OH)_2_ group and the highest cell attachment percentage was 95.08% ±1.44 in the negative control group. AgNPs gel groups showed cell attachment percentage of 93.0% ±2.52, 93.25% ±2.68 and 93.25% ±2.63 in 0.01% AgNPs group, 0.015% AgNPs group and 0.02% AgNPs group respectively. However, there was no significant difference in cell attachment between the different groups (*P* = 0.012). (Fig. 3).

**Fig. 4:**
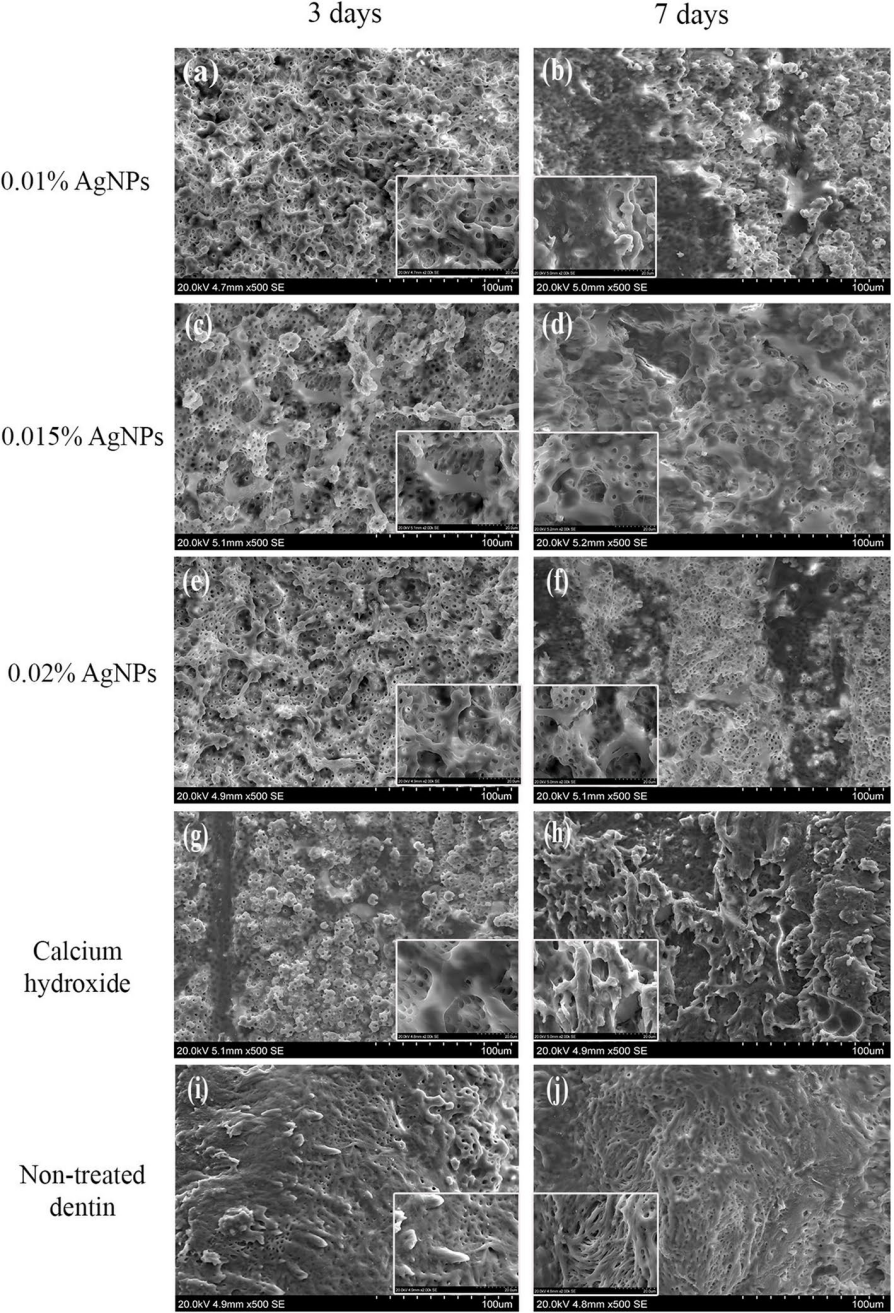
Representative SEM images showing DPSCs adhesion to the dentin surfaces in 0.01% AgNPs (**a**), 0.015% AgNPs (**b**), 0.02% AgNPs (**e**), Ca (OH)_2_ (**g**), Non-treated dentin (**i**) after 3 days and 0.01% AgNPs (**b**), 0.015% AgNPs (**d**), 0.02% AgNPs (**f**), Ca (OH)_2_ (**h**), Non-treated dentin (**j**) after 7 days of culture. Magnifications are × 500. Insets in each image represent higher magnification at ×2000 showing dense cell attachment on the dentin surfaces in all groups

Representative SEM images demonstrated DPSCs adhesion and attachment on dentin surfaces in all groups. Spindle shaped cells with cell-cell contacts and extended processes onto the dentin surface were observed (Fig. 4).

### Cytotoxicity of AgNPs

**Fig. 5:**
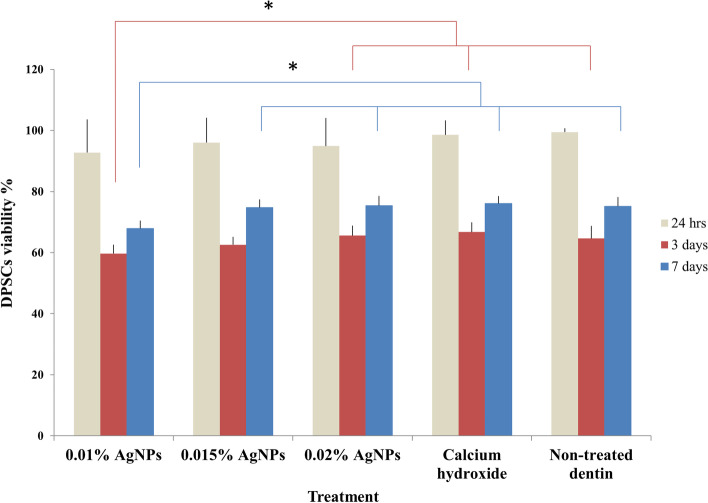
The percentages of DPSCs viability (mean ± SD) in different treatment and control groups: namely 0.01% AgNPs, 0.015% AgNPs, 0.02% AgNPs, Ca (OH)_2_ and non-treated dentin. Percentages were indirectly calculated from the cytotoxicity percentage result of the LDH assay. Experiments were repeated from 3 different donors and in triplicates (final *n* = 9). *p < 0.05

Cell viability percentage was maintained above 92.74% (±10.89) after 24 hours of culture with no statistical significant difference between test and control groups (*P* = 0.197). After 3 days, all groups showed a significantly higher death rate compared to 24 hours. When comparing all groups at 3 days, only the 0.01% AgNPs group showed statistically significant higher death rate (40.32% ±2.92) compared to 0.02% AgNPs group (34.46% ±3.24), Ca (OH)_2_ group (33.26% ±3.19) and negative control group (35.36% ±4.06) but it was not significantly different from the 0.015% AgNPs group (37.43% ±2.61). All groups recovered comparably after 7 days, except the 0.01% AgNPs group which again showed a significantly higher death rate (32.11% ±2.55) compared to all other groups (Table 1) (Fig. 5). Results on a per donor basis are presented in the supplementary information (Supplementary Table 1).

**Table 1:** Cytotoxicity of AgNPs in the study groups after 24 hours, 3 and 7 days

		AgNPs 0.01% (***n*** = 18)	AgNPs 0.015% (***n*** = 18)	AgNPs 0.02% (***n*** = 18)	Ca (OH)_**2**_ (***n*** = 18)	No treatment (***n*** = 18)	Test ***(P*** value)
24 hours	Mean (SD)	7.26 (10.89)	3.99 (8.10)	5.13 (9.22)	1.44 (4.69)	0.54 (1.24)	*X*^2^ = 6.032 (0.197)
	Median (IQR)	1.00 (21.60)	0.78 (1.93)	0.52 (7.50)	−0.11 (1.99)	0.51 (1.45)	
3 days	Mean (SD)	40.32 (2.92)^a,b^	37.43 (2.61)^b,c^	34.46 (3.24)^c^	33.26 (3.19)^c^	35.36 (4.06)^c^	F = 6.557 (< 0.001*)
	Median (IQR)	40.80 (4.66)	38.22 (4.90)	33.49 (4.05)	32.49 (4.85)	35.44 (7.25)	
7 days	Mean (SD)	32.11 (2.55)^a^	25.26 (2.63)^b^	24.61 (3.21)^b^	23.96 (2.44)^b^	24.88 (3.09)^b^	F = 2.897 (< 0.001*)
	Median (IQR)	32.01 (4.65)	25.66 (3.74)	23.98 (5.15)	23.39 (4.37)	25.35 (4.77)	
**Test (*(P*** **value)**		*X*^2^ = 29.398 (< 0.001*)	*X*^2^ = 27.657 (< 0.001*)	*X*^2^ = 28.209 (< 0.001*)	*X*^2^ = 29.562 (< 0.001*)	*X*^2^ = 28.418 (< 0.001*)	
**Pair wise comparisons**		*P*_*1*_ < 0.001*	*P*_*1*_ < 0.001*	*P*_*1*_ < 0.001*	*P*_*1*_ < 0.001*	*P*_*1*_ < 0.001*	
		*P*_*2*_ = 0.005*	*P*_*2*_ = 0.006*	*P*_*2*_ = 0.009*	*P*_*2*_ = 0.005*	*P*_*2*_ = 0.004*	
		*P*_*3*_ *= 0.231*	*P*_*3*_ *= 0.228*	*P*_*3*_ *= 0.181*	*P*_*3*_ *= 0.210*	*P*_*3*_ *= 0.263*	

*Statistically significant different at *p* value≤0.05

^a,b,c^ different letters denote statistically significant difference between groups at each time point

*P*_*1:*_ comparison between 24 hrs and 3 days, *P*_*2:*_ comparison between 24 hrs and 7 days, *P*_*3:*_ comparison between 3 days and 7 days within each group

### DPSCs viability

**Fig. 6:**
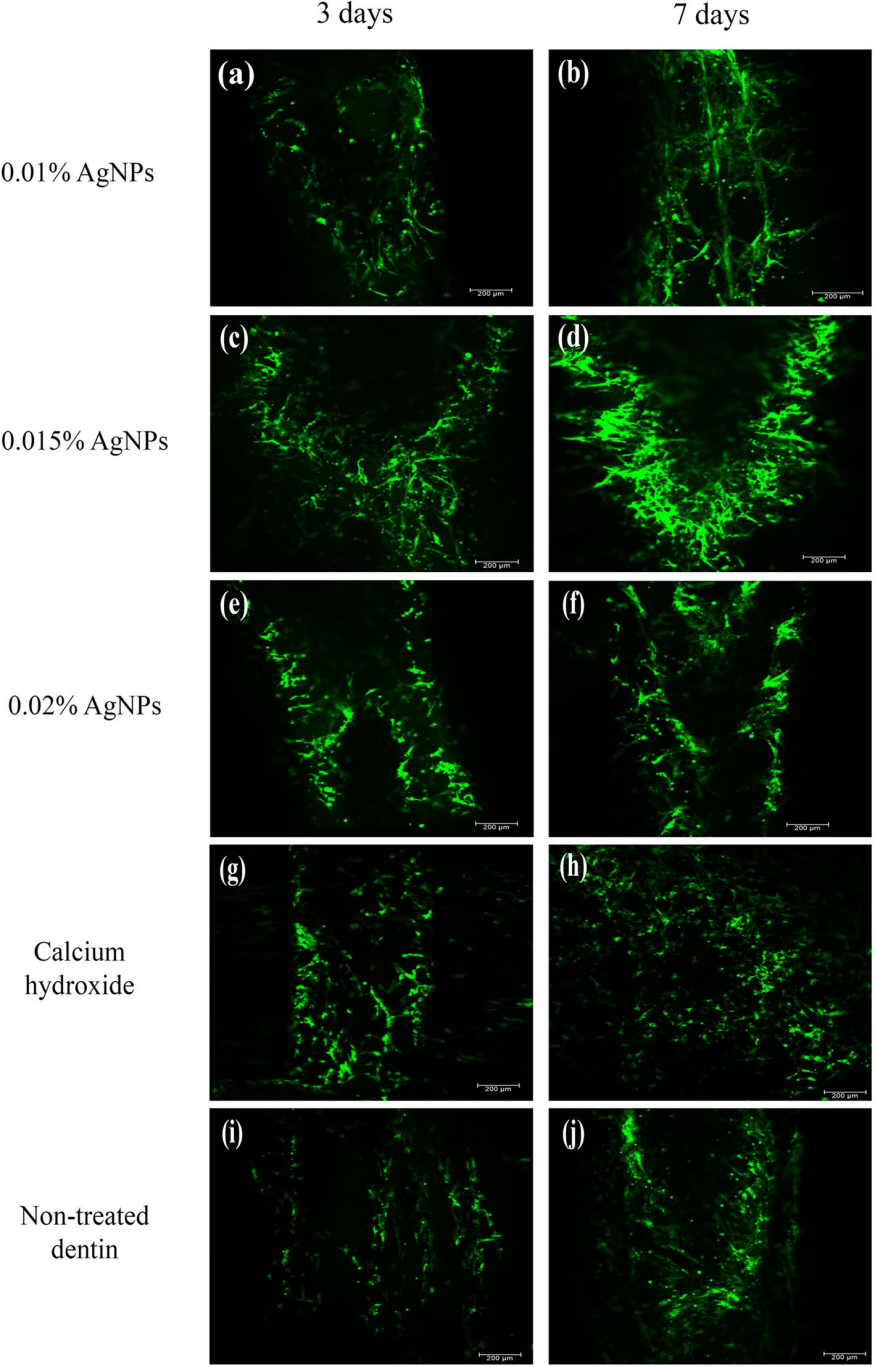
Confocal microscopic images showing DPSCs viability assessed by LIVE/DEAD staining surfaces in 0.01% AgNPs (**a**), 0.015% AgNPs (**c**), 0.02% AgNPs (**e**), Ca (OH)_2_ (**g**), Non-treated dentin (**i**) after 3 days and 0.01% AgNPs (**b**), 0.015% AgNPs (**d**), 0.02% AgNPs (**f**), Ca (OH)_2_ (h), Non-treated dentin (j) after 7 days of culture. The raw images were acquired at a resolution of 72 dpi while the final figure was saved in TIF format with a resolution of 284 dpi. (Scale bar 200 μm)

All groups showed viable DPSCs (stained in green) growing on the pulp space and lumen of the dentin discs with minimal number of dead cells (stained in red). DPSCs maintained a spindle-shaped, fibroblast like cell morphology in all groups (Fig. 6). Multiple supporting fields of cells are shown as supplementary information (Supplementary Fig. 2).

### DPSCs proliferation on dentin disc surface

**Fig. 7:**
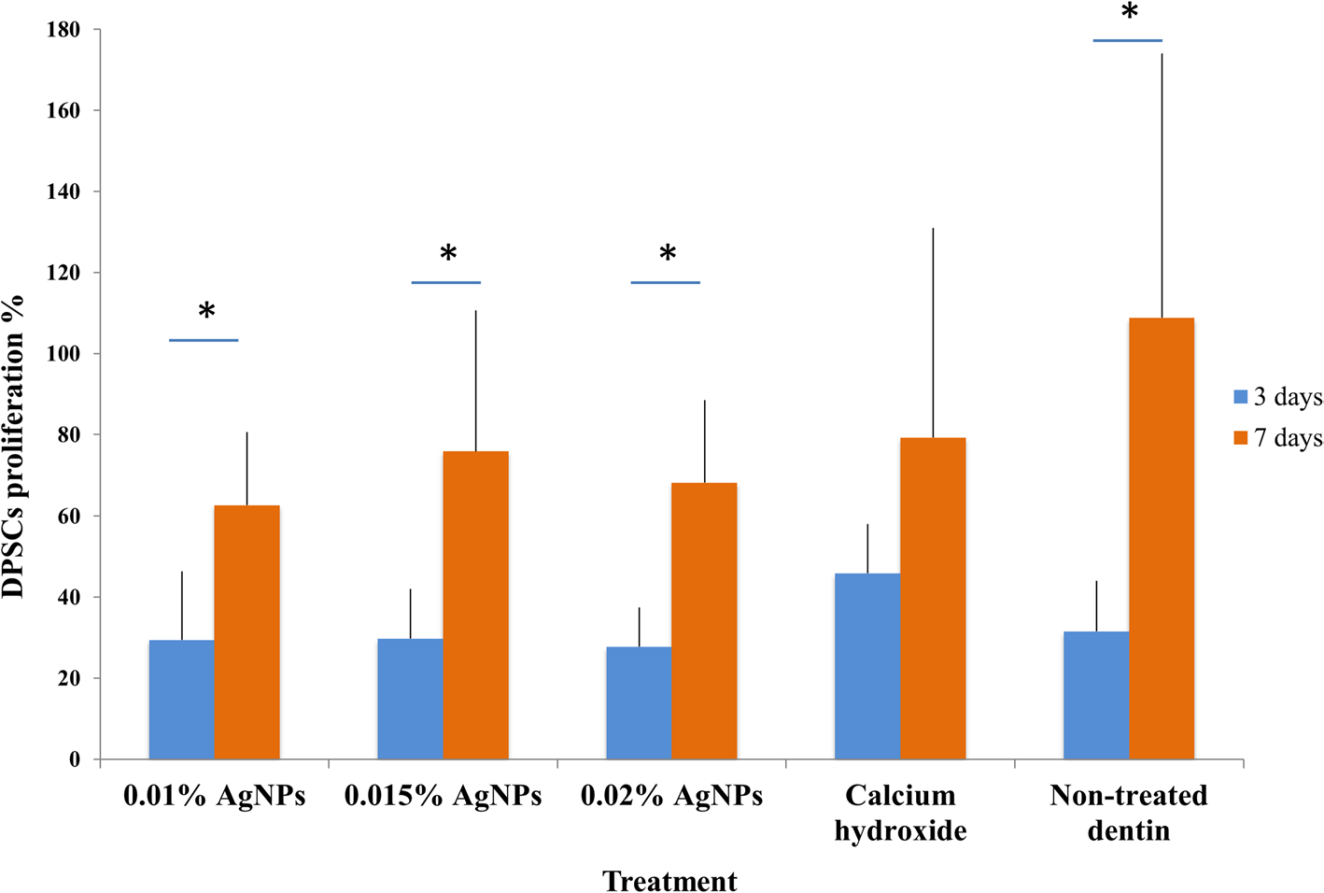
Assessment of DPSCs proliferation rate after seeding on treated dentin surfaces showing the mean ± SD of the following treatment and control groups: 0.01% AgNPs, 0.015% AgNPs, 0.02% AgNPs, Ca (OH)_2_, non-treated dentin, after 3 and 7 days of culture. Experiments were repeated from 3 different donors and in triplicates (final *n* = 9) for each group. *p < 0.05

DPSCs proliferation at 3 and 7 days was compared in all groups using the WST-1 assay and were normalized to the absorbance values of the initial seeding density of 10 × 10^3^ cells. DPSCs proliferation percentages nearly doubled from 3 days to 7 days in all AgNPs concentrations groups which were statistically significant however; there was no statistical significant difference between different groups at 3 days or at 7 days. Furthermore, the negative control group showed a massive increase in cell proliferation from 31.56% ±12.49 at 3 days to 108.81% ±65.27 at 7 days that was statistically significant. However, the increase in proliferation in Ca (OH)_2_ group was not statistically significant between 3 days (45.89% ±12.12) and 7 days (79.25% ±51.68) (Fig. 7). Results on a per donor basis are presented in the supplementary information (Supplementary Fig. 3-5 and supplementary Table 2).

## Discussion

Many studies tested the antimicrobial effect of AgNPs as an antimicrobial agent and its cytotoxic effect on different cell lines in many biomedical and textile contexts [35] but to our knowledge, so far the cytotoxic effect of AgNPs on DPSCs for usage as intra-canal medicament during REPs has not been investigated. The antimicrobial effects of AgNPs gels and their numerous applications have been widely reported in the literature [25]. In the field of endodontics, Wu et al. evaluated the antimicrobial effect of AgNPs as an irrigant and intra-canal medicament and concluded that 0.02% AgNPs gel as a medicament significantly disrupted the biofilm and resulted in the least number of post-treatment residual viable *E. faecalis* compared to Ca (OH)_2_ [26]. Furthermore, 0.02% AgNPs gel proved to maintain effective antimicrobial properties up to 24 hours post application [27]. However, to our knowledge, the cytocompatibility of AgNPs against DPSCs has not yet been investigated. Hence, the current study aimed to investigate the cytocompatibility of AgNPs gel for potential use as future intra-canal medicament for regenerative endodontic applications. It has been shown that dentin conditioning is crucial for determining stem cell fate in addition to providing adequate disinfection [19]. Stem cell fate is also critical if the tissues regenerated will eventually simulate the actual lost/damaged tissues. The protocols for dentin conditioning have been shown to influence growth factor release and hence cell behavior [36]. In the present study Ca (OH)_2_ was chosen as the positive control since it has been shown that Ca (OH)_2_ promoted cell attachment to dentin discs [37], had no cytotoxic effects on stem cells of the apical papilla (SCAP) [18, 19] and promoted stem cell survival and growth [38]. In an attempt to simulate the clinical situation, the current study used dentin discs and DPSCs which may survive despite the development of peri-radicular infection which can destroy the stem cells at the apical papilla. The rich blood supply through the wide open apex, and even in mature teeth, may guarantee the possibility of remaining vital pulp tissues when a peri-radicular lesion has developed hence the choice of this cell population [39, 40]. Furthermore, DPSCs have been previously used by many other studies to investigate the effects of different materials on the biological responses of human stem cells [41, 42].

In the current study, complete removal of AgNPs gel was achieved after our irrigation protocol while residual Ca (OH)_2_ particles were still attached to the dentin surface in accordance with a previous study demonstrating that Ca (OH)_2_ remained in the dentin even after root canal irrigation [43]. This illustrates an advantage of easy removal of AgNPs gel compared to Ca (OH)_2_.

For REPs, ideally, cells should migrate into the root canal and attach to the dentin surface to differentiate into odontoblast-like cells and be able to regenerate damaged dentin-pulp complex [44]. Additionally, maintenance of dentin matrix mechanical properties is important because it influences cell differentiation [45].

Although our dentin sterilization protocol has been previously established in other studies [37], there were some concerns that this approach might negatively affect the extra cellular dentinal proteins and growth factors. However, it has been demonstrated that treated dentin matrix (TDM) maintained its biocompatibility and promoted differentiation of DPSCs after autoclaving [46], which was also confirmed in this study in all groups.

In this study, we found that all concentrations of AgNPs promoted cell attachment to dentin discs comparable to Ca (OH)_2_. Favorable stem cell attachment in response to AgNPs formulations has also been documented by several other studies [47, 48].

There have been concerns regarding cytotoxicity of AgNPs, which is mainly related to the release of free Ag+. Greulich et al., found that 100 nm sized PVP-coated AgNPs showed cytotoxicity to human mesenchymal stem cells (hMSCs) at AgNPs concentrations above 5 μg/mL after 7 days [49] and Pauksch et al. demonstrated that cell viability of hMSCs was impaired after treatment at a concentration of 10 μg/g for 21 days [50]. The results of our study showed that while there was an initial effect on cell viability which may be attributed to the residual effect of the irrigation protocol used, cells began to recover after 7 days indicating that initial cytotoxic effect was transient. Others have shown that the effect of NaOCL is concentration dependant and its negative effect can be reversed by application of 17% EDTA after 7 days [51]. In the context of REPs, this coincides with the minimum recommended time for application of intra-canal medicaments which is 1 week [2]. Hence, after a 7-day treatment, cells would hypothetically be able to attach and proliferate on dentin treated with 0.02% AgNPs gel which was found to effectively disrupt *E. faecalis* biofilms [27]. However, the difference in our results compared to previous studies may owe to particle size, concentration, the formulation used and method of fabrication of AgNPs. Indeed, it has been shown that AgNPs biocompatibility is concentration dependent with increased cytotoxicity at higher concentrations [52, 53]. Surprisingly, in our study the lower concentration of (0.01%) AgNPs showed some significant cytotoxic effect at 3 and 7 days while the higher concentrations (0.015 and 0.02%) of AgNPs showed no significant cytotoxic effect (less than 40%) compared to control groups. This may be because of higher concentrations of AgNPs tend to aggregate the nanoparticles. This may lead to lower cytotoxicity because the aggregated nanoparticles are of a size that cannot cross the cell membrane. On the other hand, aggregates can still exhibit the antibacterial effect [54, 55]. This result indicates that, in spite of the cytotoxic effect of this medicament an almost 60% cell viability was maintained which may be sufficient for tissue regeneration [56].

This result is in accordance with Samberg et al. who demonstrated that exposure of human adipose derived stem cells to 10 and 20 nm AgNPs resulted in no significant cytotoxicity [57]. However, Alt et al. concluded that 1% of nano-silver loaded cement showed no cytotoxicity to human osteoblasts [58]. Previously mentioned studies confirmed that, the variability on reported toxicity of AgNPs depends not only on nanoparticles concentration and incubation time, but also on other factors such as nanoparticles’ size, synthesis method, duration of particle storage and experimental design.

We have found that DPSCs were able to significantly proliferate between 3 and 7 days of culture highlighting that, there was no long-term detrimental effect of the residual material from the different concentrations of AgNPs. This confirmed that in spite of a relatively high initial cytotoxic effect of all groups, DPSCs were able to recover and increase in number. These findings are consistent with the observations of Chang et al. who found that, mesenchymal stem cells harvested from human umbilical cord Wharton’s jelly tissue, were able to proliferate in presence of AgNPs [48]. Liu et al. illustrated that hMSCs numbers increased after treatment with different concentrations of AgNPs [59].

However, while previously mentioned studies used lower concentrations of AgNPs compared to the concentrations we used, they tested the direct effect of AgNPs while we tested the indirect effect by culturing DPSCs on the dentin surface previously treated with AgNPs which may explain the cytocompatibility of our higher concentrations of AgNPs compared to controls. It is noteworthy that in a clinical scenario during the induction of bleeding step, cells would not come in direct contact with the intra-canal medicament but rather be affected by the residual and lingering effect of the medicament following the final rinse protocol [60]. More research is required to test the long term effect of residual AgNPs at longer time points in-vitro and in-vivo. The use of our current preparation of AgNPs gel seem to offer obvious advantages over the traditional Ca (OH)_2_, minimizing re-infection of root canals [61], offering both easier infiltration inside the dentinal tubules and lateral canals [62] and easier removal and clearing from the dentin surface. Additionally, AgNPs can be designed through green synthesis and echo friendly approaches minimizing carbon foot print [63]. The stability of the current AgNPs preparation eliminates the need for re-application, unlike Ca (OH)_2_ which needs to be reapplied to maintain acceptable levels of disinfection.

One of the limitations of this study was that we did not test the effect of different particle sizes of AgNPs. Furthermore, we did not test the cytotoxic effect of AgNPs against SCAP and other cell populations that are targeted during REPs and thus further studies are required. We have also faced a technical challenge with the dentin discs because the presence of the lumen created a 2 plane surface which made it difficult to control and standardize the number of cells that attached to the lumen, and those attaching to the other plane in the dentin. Having said that, the presence of different planes including the pulp canal lumen was more representative of the clinical situation. Silver is known to cause black staining of dental tissues in certain chemical formulations such as the Silver diamine fluoride (SDF) which has been used as an inexpensive and easy to apply cariostatic agent to dentin [64]. However, other formulations such as adding AgNPs to sodium fluoride (NaF) solution showed no tooth-staining effect [65]. Furthermore, applying the AgNPs gel as an intra-canal medicament is a different situation where the medicament will be removed after 7 days, reducing the possibility of staining. However further investigation into the chemical formulation that is suitable as a medicament requires further investigation.

## Conclusion

Dentin discs treated for 7 days with concentrations of AgNPs gel ranging from 0.01–0.02% allowed more than 90% DPSCs cell attachment after 24 hours. Furthermore, the cytotoxicity and proliferation of DPSCs in response to AgNPs gel were comparable to those with calcium hydroxide. These results suggest that AgNPs gel may represent a promising future candidate for clinical use in regenerative endodontics. However, its effects may be concentration-dependent warranting further investigation.

## Supplementary Information

**Additional file 1: Supplementary Fig. 1.** AgNPs gel loaded in insulin syringe.

**Additional file 2: Supplementary Fig. 2.** Confocal microscopic images showing DPSCs viability assessed by LIVE/DEAD staining surfaces in 0.01% AgNPs (a), 0.015% AgNPs (c), 0.02% AgNPs (e), Ca (OH)_2_ (g), Non-treated dentin (i) after 3 days and 0.01% AgNPs (b), 0.015% AgNPs (d), 0.02% AgNPs (f), Ca (OH)_2_ (h), Non-treated dentin (j) after 7 days of culture. The raw images were acquired at a resolution of 72 dpi while the final figure was saved in TIF format with a resolution of 284 dpi. (Scale bar 200 μm).

**Additional file 3: Supplementary Fig. 3.** Assessment of DPSCs proliferation rate on dentin surfaces in treatment and control groups in donor 1: 0.01% AgNPs, 0.015% AgNPs, 0.02% AgNPs, Ca (OH)_2_ and non-treated dentin, after 3 and 7 days of culture.

**Additional file 4: Supplementary Fig. 4.** Assessment of DPSCs proliferation rate on dentin surfaces in treatment and control groups in donor 2: 0.01% AgNPs, 0.015% AgNPs, 0.02% AgNPs, Ca (OH)_2_ and non-treated dentin, after 3 and 7 days of culture.

**Additional file 5: Supplementary Fig. 5.** Assessment of DPSCs proliferation rate on dentin surfaces in treatment and control groups in donor 3: 0.01% AgNPs, 0.015% AgNPs, 0.02% AgNPs, Ca (OH)_2_ and non-treated dentin, after 3 and 7 days of culture.

**Additional file 6: Supplementary Table 1.** Comparison of cytotoxicity between the study groups on donor basis. **Supplementary Table 2.** Comparison of proliferation rates between the study groups on donor basis.

## Data Availability

The data and materials collected in this research are available from the corresponding author when requested reasonably.

## Abbreviations

REPs: Regenerative Endodontic Procedures
TAP: Triple Antibiotic Paste
DAP: Double Antibiotic Paste
Ca (OH)_2_: Calcium Hydroxide
AgNPs: Silver Nanoparticles
ROS: Reactive Oxygen Species
GSH: Glutathione
LDH: Lactate Dehydrogenase
DPSCs: Human Dental Pulp Stem Cells
AgNO_3_: Silver Nitrate
PVP: Polyvinylpyrrolidone
SEM: Scanning Electron Microscopy
α-MEM: Alpha Modified Eagle’s Minimum Essential Medium
EthD-1: Ethidium Homodimer
SCAP: Stem Cells of the Apical Papilla
TDM: Treated Dentin Matrix
hMSCs: Human Mesenchymal Stem Cells
SDF: Silver Diamine Fluoride
NaF: Sodium Fluoride
