# Supplementary figures and images for: Investigating the residual effect of silver nanoparticles gel as an intra-canal medicament on dental pulp stromal cells
Source: BMC Oral Health. 2022 Nov 30;22:545. doi: 10.1186/s12903-022-02542-2 (PMC9710138; doi:10.1186/s12903-022-02542-2)

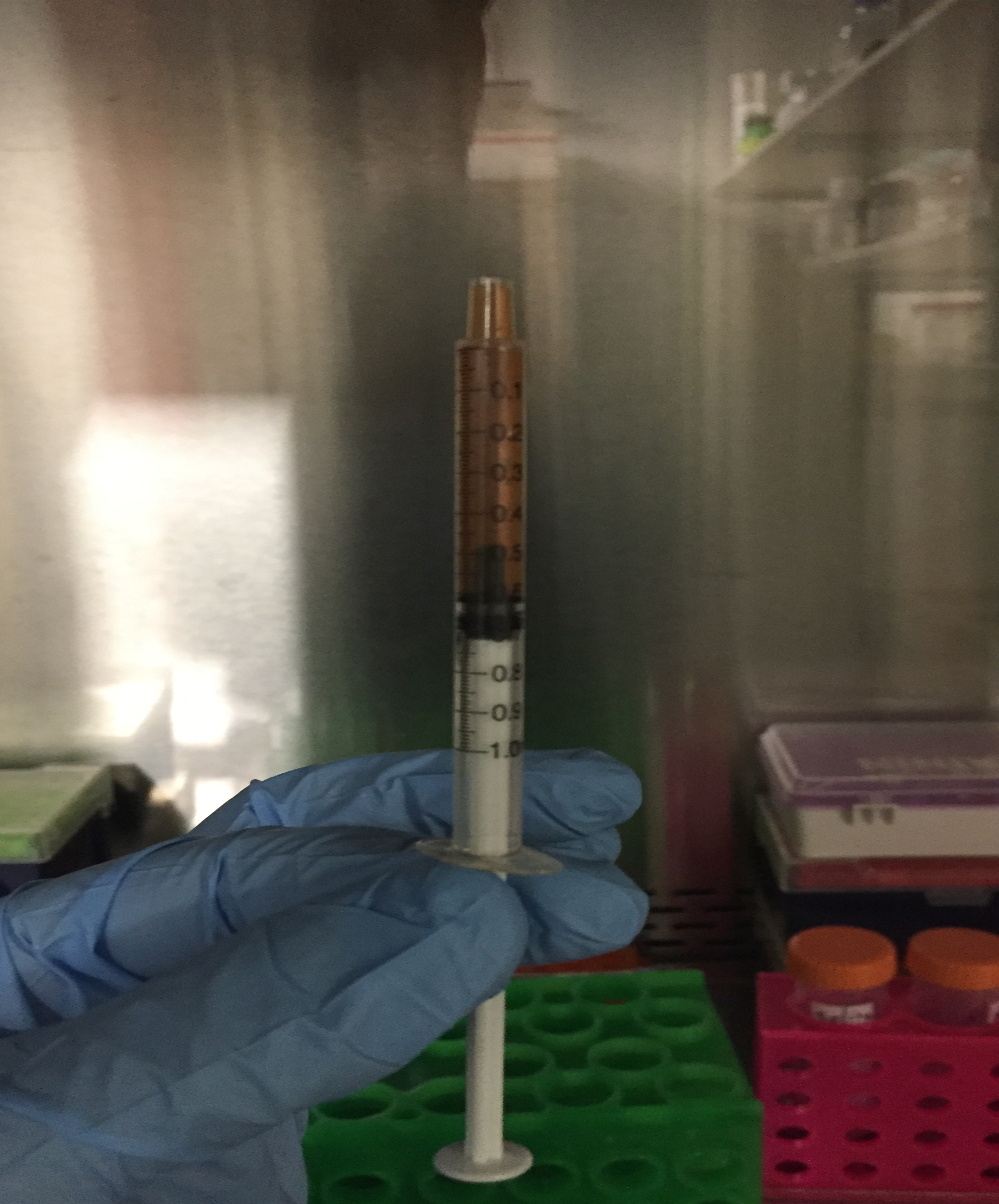

Supplement: Supplementary file 1 — Additional file 1: Supplementary Fig. 1. AgNPs gel loaded in insulin syringe. [file 12903_2022_2542_MOESM1_ESM.tif]

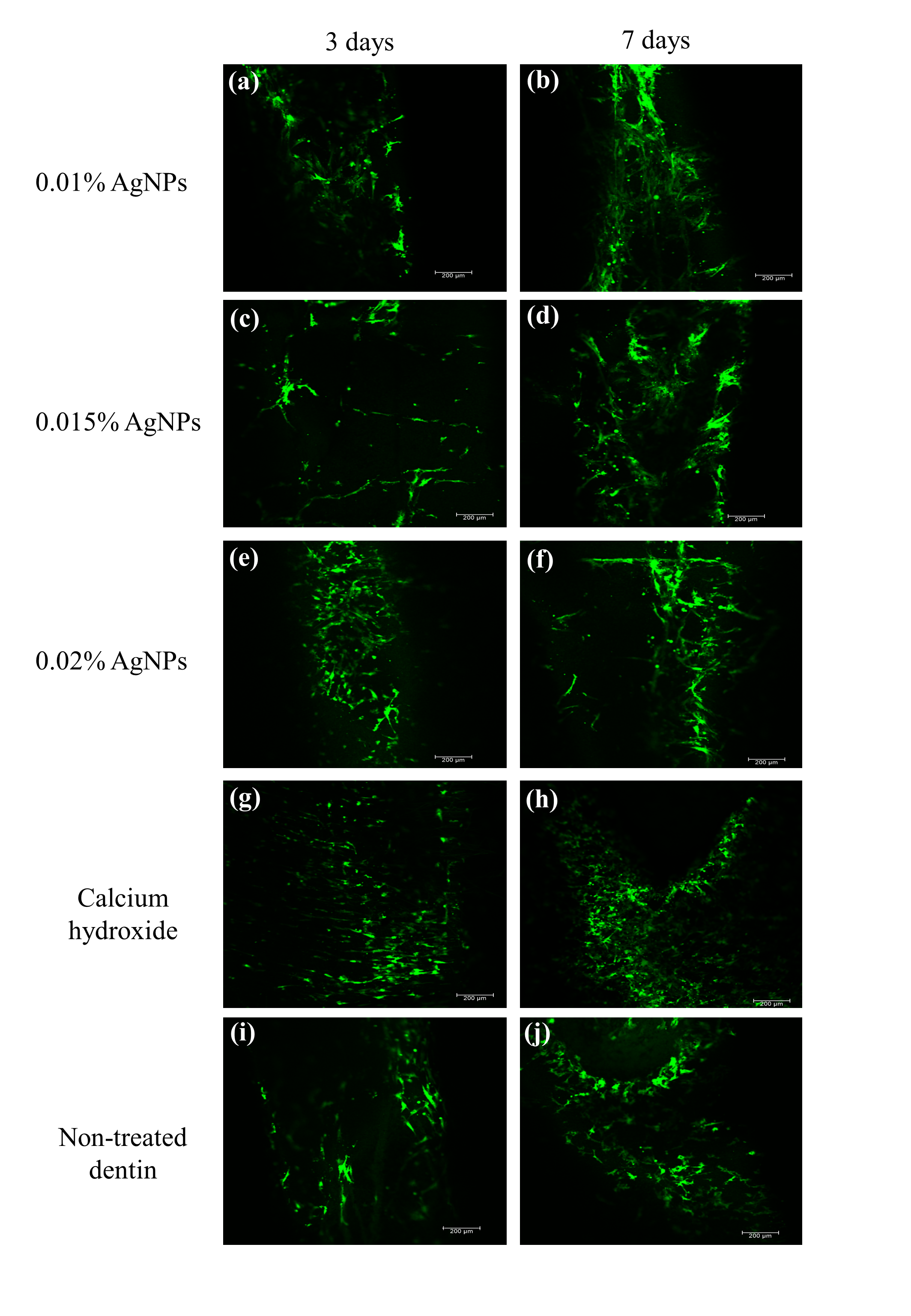

Supplement: Supplementary file 2 — Additional file 2: Supplementary Fig. 2. Confocal microscopic images showing DPSCs viability assessed by LIVE/DEAD staining surfaces in 0.01% AgNPs (a), 0.015% AgNPs (c), 0.02% AgNPs (e), Ca (OH)2 (g), Non-treated dentin (i) after 3 days and 0.01% AgNPs (b), 0.015% AgNPs (d), 0.02% AgNPs (f), Ca (OH)2 (h), Non-treated dentin (j) after 7 days of culture. The raw images were acquired at a resolution of 72 dpi while the final figure was saved in TIF format with a resolution of 284 dpi. (Scale bar 200 μm). [file 12903_2022_2542_MOESM2_ESM.tif]

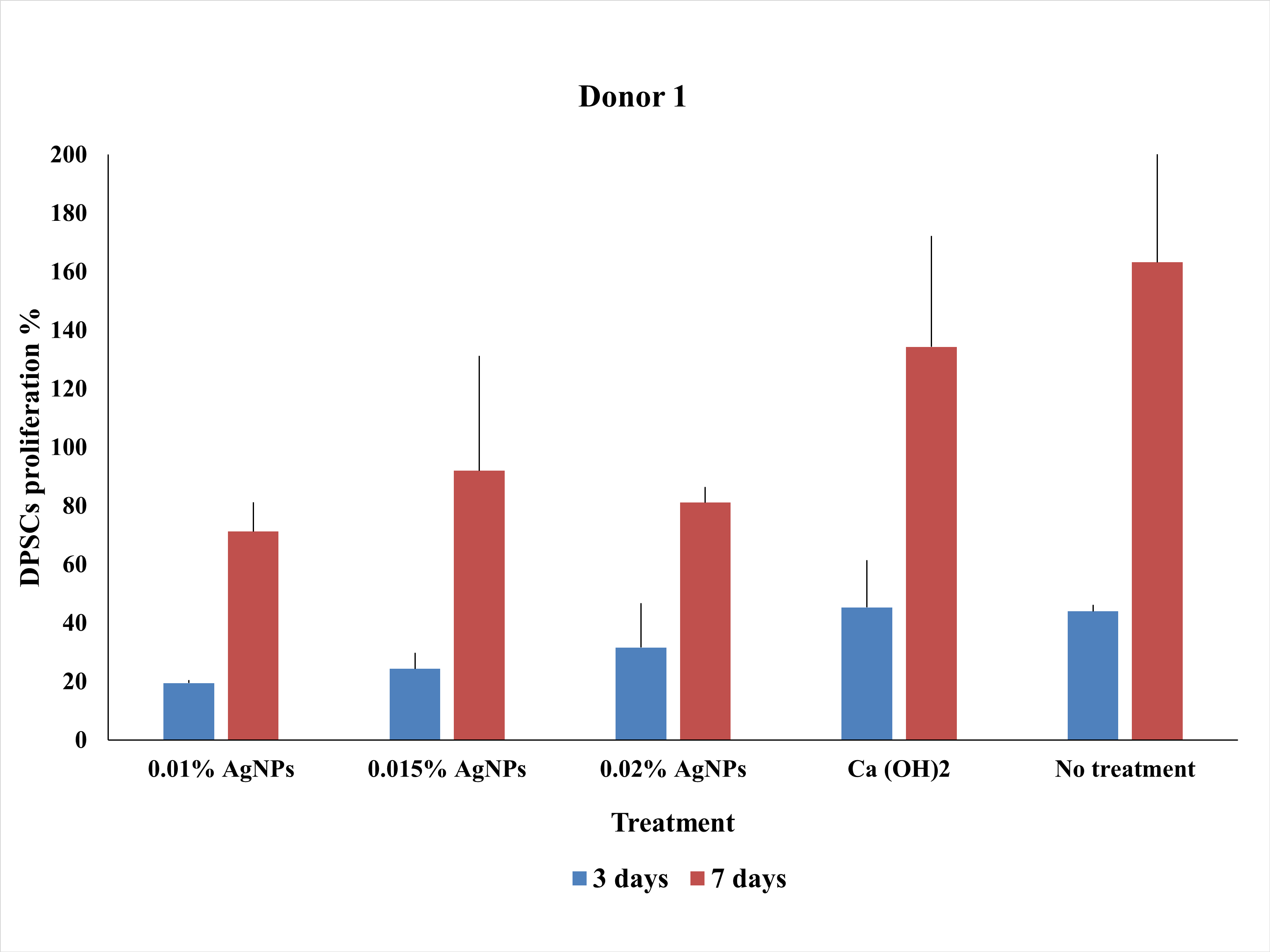

Supplement: Supplementary file 3 — Additional file 3: Supplementary Fig. 3. Assessment of DPSCs proliferation rate on dentin surfaces in treatment and control groups in donor 1: 0.01% AgNPs, 0.015% AgNPs, 0.02% AgNPs, Ca (OH)2 and non-treated dentin, after 3 and 7 days of culture. [file 12903_2022_2542_MOESM3_ESM.tif]

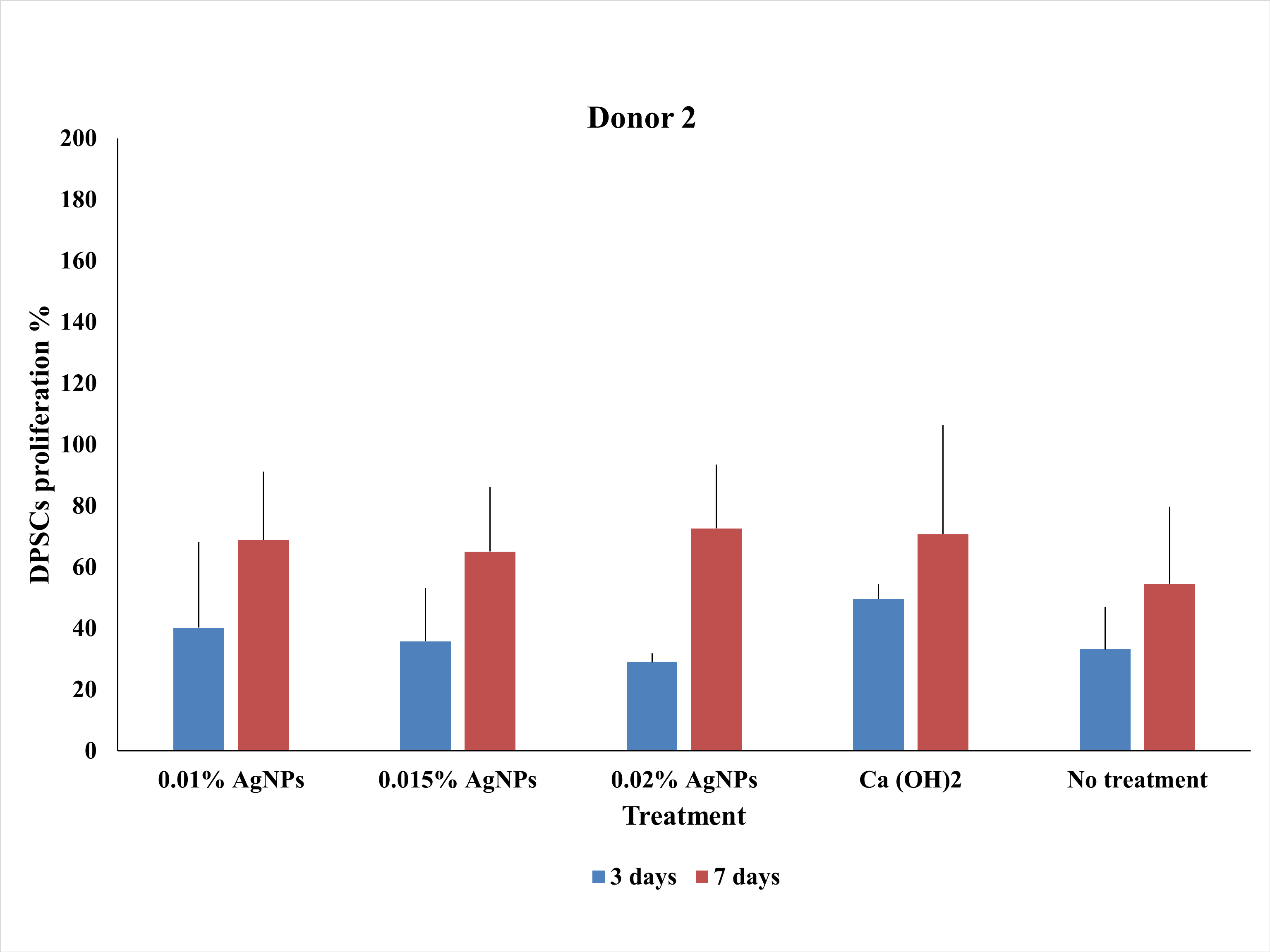

Supplement: Supplementary file 4 — Additional file 4: Supplementary Fig. 4. Assessment of DPSCs proliferation rate on dentin surfaces in treatment and control groups in donor 2: 0.01% AgNPs, 0.015% AgNPs, 0.02% AgNPs, Ca (OH)2 and non-treated dentin, after 3 and 7 days of culture. [file 12903_2022_2542_MOESM4_ESM.tif]

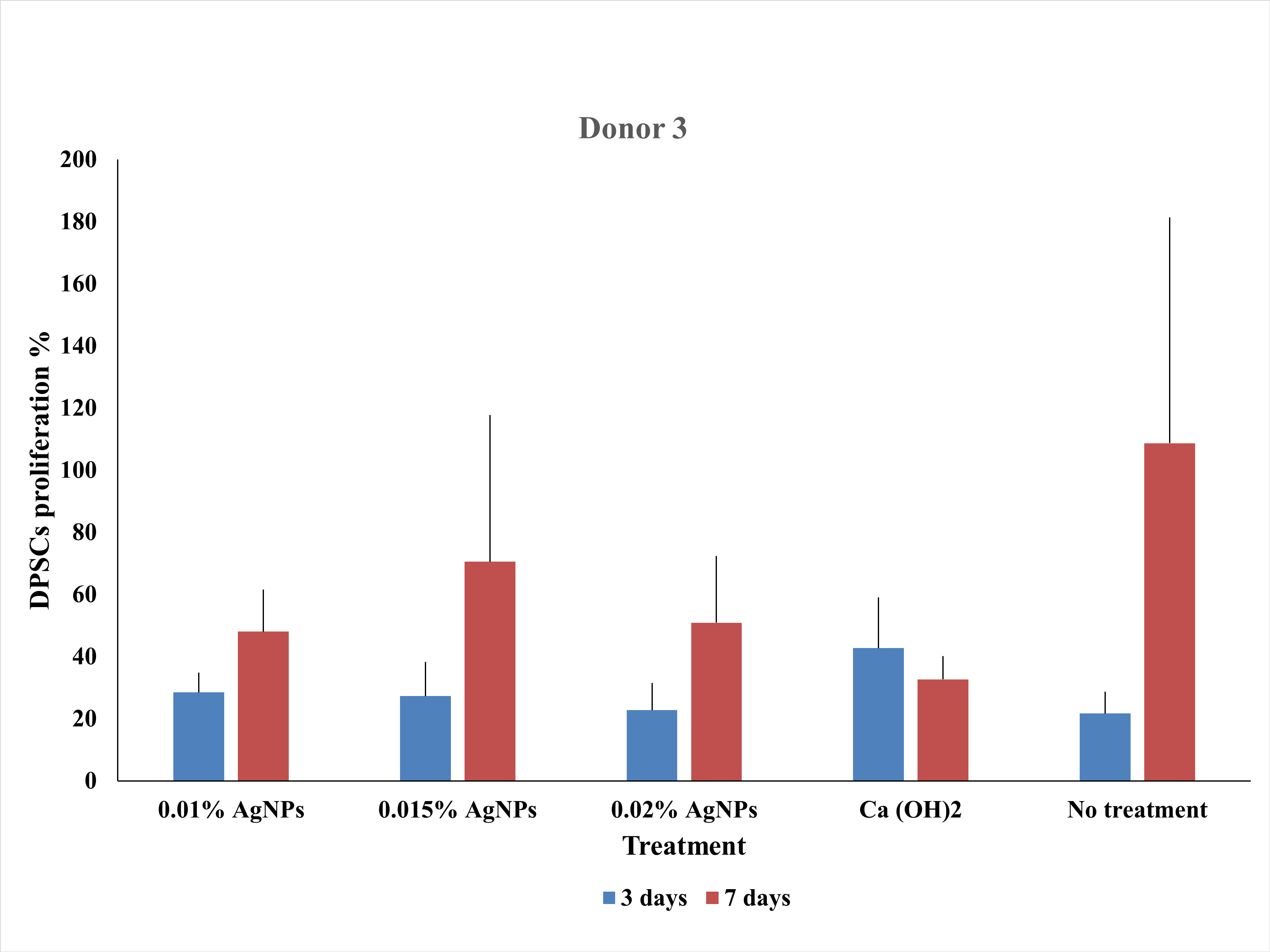

Supplement: Supplementary file 5 — Additional file 5: Supplementary Fig. 5. Assessment of DPSCs proliferation rate on dentin surfaces in treatment and control groups in donor 3: 0.01% AgNPs, 0.015% AgNPs, 0.02% AgNPs, Ca (OH)2 and non-treated dentin, after 3 and 7 days of culture. [file 12903_2022_2542_MOESM5_ESM.tif]
