# Supplementary material for: Investigating the residual effect of silver nanoparticles gel as an intra-canal medicament on dental pulp stromal cells
Source: BMC Oral Health. 2022 Nov 30;22:545. doi: 10.1186/s12903-022-02542-2 (PMC9710138; doi:10.1186/s12903-022-02542-2)
Supplement: Supplementary file 6 — Additional file 6: Supplementary Table 1. Comparison of cytotoxicity between the study groups on donor basis. Supplementary Table 2. Comparison of proliferation rates between the study groups on donor basis. [file 12903_2022_2542_MOESM6_ESM.docx]

**Supplementary table 1: Comparison of cytotoxicity between the study groups on donor basis:**

|  | | | **0.01% AgNPs** | **0.015% AgNPs** | **0.02% AgNPs** | **Ca (OH)2** | **No treatment** | **P value** |
| --- | --- | --- | --- | --- | --- | --- | --- | --- |
| **Donor 1** | 24 hours | Mean (SD) | 16.64 (12.95) | 0.26 (0.83) | 7.40 (11.26) | 0.03 (0.50) | 0.44 (0.85) | 0.197 |
|  |  | Median (Range) | 24.05 (27.33)^A^ | 0.37 (1.95) | 0.53 (23.11)^A^ | -0.18 (1.22)^A^ | 0.64 (2.45) |  |
|  | 3 days | Mean (SD) | 39.17 (2.54) | 33.68 (0.39) | 31.63 (2.06) | 30.41 (2.09) | 30.48 (1.11) | **0.049*** |
|  |  | Median (Range) | 39.39 (5.06)^AB^ | 33.68 (0.55) | 32.47 (3.85)^AB^ | 31.58 (3.65)^AB^ | 30.48 (1.57) |  |
|  | 7 days | Mean (SD) | 32.74 (2.61) | 26.16 (0.74) | 26.25 (1.97) | 25.79 (2.88) | 26.45 (1.89) | 0.145 |
|  |  | Median (Range) | 32.02 (5.06)^B^ | 25.81 (1.35) | 27.32 (3.47)^B^ | 27.27 (5.17)^B^ | 25.51 (3.42) |  |
|  | **P value** | | **0.050*** | 0.135 | **0.050*** | **0.050*** | 0.135 |  |
| **Donor 2** | 24 hours | Mean (SD) | 0.91 (0.94) | 0.78 (1.02) | 0.73 (1.45) | 0.66 (1.29) | 0.84 (2.03) | 0.980 |
|  |  | Median (Range) | 0.49 (2.42)^A^ | 0.73 (2.06)^A^ | 0.69 (3.05)^A^ | 0.80 (2.81)^A^ | 0.60 (4.77)^A^ |  |
|  | 3 days | Mean (SD) | 42.01 (1.05) | 39.15 (0.44) | 36.87 (3.52) | 32.81 (2.41) | 34.60 (1.78) | **0.025*** |
|  |  | Median (Range) | 42.57 (1.86)^a,AB^ | 39.12 (0.44)^ab,AB^ | 36.58 (7.03)^ab,AB^ | 32.49 (4.79)^b,AB^ | 34.43 (3.54)^ab,AB^ |  |
|  | 7 days | Mean (SD) | 30.23 (2.52) | 24.44 (2.00) | 21.73 (1.66) | 22.43 (2.02) | 23.41 (3.47) | 0.077 |
|  |  | Median (Range) | 29.39 (4.82)^B^ | 25.50 (3.54)^B^ | 22.03 (3.29)^B^ | 21.86 (3.91)^B^ | 24.53 (6.67)^B^ |  |
|  | **P value** | | **0.050*** | **0.050*** | **0.050*** | **0.050*** | **0.050*** |  |
| **Donor 3** | 24 hours | Mean (SD) | 4.26 (8.39) | 10.95 (11.60) | 7.26 (11.21) | 3.65 (8.01) | 0.34 (0.48) | 0.497 |
|  |  | Median (Range) | 1.01 (21.57)^A^ | 10.37 (22.23)^A^ | 0.42 (22.33)^A^ | 0.19 (20.14)^A^ | 0.49 (1.20)^A^ |  |
|  | 3 days | Mean (SD) | 39.79 (4.48) | 38.23 (2.06) | 34.91 (2.12) | 36.59 (1.27) | 39.39 (2.25) | 0.305 |
|  |  | Median (Range) | 40.56 (8.86)^AB^ | 37.73 (4.04)^AB^ | 35.71 (4.00)^AB^ | 36.47 (2.54)^AB^ | 39.54 (4.49)^AB^ |  |
|  | 7 days | Mean (SD) | 33.38 (2.16) | 25.20 (4.58) | 25.84 (4.01) | 23.67 (1.69) | 24.80 (3.97) | 0.124 |
|  |  | Median (Range) | 34.55 (3.82)^B^ | 25.82 (9.10)^B^ | 24.82 (7.82)^B^ | 23.40 (3.34)^B^ | 26.08 (7.62)^B^ |  |
|  | **P value** | | **0.050*** | **0.050*** | **0.050*** | **0.050*** | **0.050*** |  |

*Statistically significant difference at p value<0.05

Different statistical superscript lowercase letters denote statistically significant differences between groups

Different statistical superscript uppercase letters denote statistically significant differences within groups

**Supplementary table 2: Comparison of proliferation rates between the study groups on donor basis:**

|  | | | **0.01% AgNPs** | **0.015% AgNPs** | **0.02% AgNPs** | **Ca (OH)2** | **No treatment** | **P value** |
| --- | --- | --- | --- | --- | --- | --- | --- | --- |
| **Donor 1** | 3 days | Mean (SD) | 19.48 (1.07) | 24.35 (5.48) | 31.66 (15.13) | 45.37 (16.12) | 43.96 (2.21) | 0.175 |
|  |  | Median (Range) | 20.02 (1.94) | 24.35 (7.75) | 31.81 (30.27) | 52.68 (29.67) | 43.96 (3.13) |  |
|  | 7 days | Mean (SD) | 71.27 (10.03) | 92.00 (39.27) | 81.16 (5.29) | 134.34 (37.94) | 163.22 (47.64) | 0.067 |
|  |  | Median (Range) | 67.44 (18.94) | 73.71 (71.87) | 81.01 (10.59) | 134.09 (75.89) | 162.87 (95.28) |  |
|  | **P value** | | **0.049*** | 0.083 | **0.049*** | **0.049*** | 0.083 |  |
| **Donor 2** | 3 days | Mean (SD) | 40.25 (28.00) | 35.79 (17.38) | 28.97 (2.99) | 49.60 (4.88) | 33.15 (13.84) | 0.448 |
|  |  | Median (Range) | 26.14 (50.40) | 43.74 (31.91) | 27.63 (5.52) | 48.06 (9.39) | 35.24 (27.44) |  |
|  | 7 days | Mean (SD) | 68.83 (22.35) | 65.06 (21.15) | 72.61 (20.82) | 70.77 (35.64) | 54.52 (25.22) | 0.920 |
|  |  | Median (Range) | 62.97 (43.54) | 66.70 (42.20) | 81.61 (38.62) | 78.77 (69.93) | 48.96 (49.50) |  |
|  | **P value** | | 0.275 | 0.275 | **0.049*** | 0.513 | 0.275 |  |
| **Donor 3** | 3 days | Mean (SD) | 28.47 (6.39) | 27.29 (11.04) | 22.76 (8.73) | 42.69 (16.35) | 21.72 (7.03) | 0.284 |
|  |  | Median (Range) | 25.39 (11.63) | 21.51 (19.68) | 17.79 (15.21) | 46.86 (31.91) | 22.42 (14.02) |  |
|  | 7 days | Mean (SD) | 48.01 (13.64) | 70.53 (47.18) | 50.89 (21.48) | 32.65 (7.46) | 108.70 (72.65) | 0.357 |
|  |  | Median (Range) | 41.20 (24.60) | 43.44 (81.86) | 61.77 (38.62) | 32.25 (14.91) | 137.08 (136.73) |  |
|  | **P value** | | **0.049*** | **0.049*** | 0.127 | 0.513 | 0.127 |  |

*Statistically significant difference at p value<0.05
